# Acute *Cytauxzoon felis* Cases in Domestic Cats from Eastern Kansas, a Retrospective Case-Control Study (2006–2019)

**DOI:** 10.3390/vetsci7040205

**Published:** 2020-12-18

**Authors:** Yvonne M. Wikander, Qing Kang, Kathryn E. Reif

**Affiliations:** 1 Department of Diagnostic Medicine/Pathobiology, College of Veterinary Medicine, Kansas State University, Manhattan, KS 66506, USA; ywikander@vet.k-state.edu; 2 Department of Statistics, College of Arts and Sciences, Kansas State University, Manhattan, KS 66506, USA; qkang@k-state.edu

**Keywords:** *Cytauxzoon felis*, cytauxzoonosis, domestic cat, fatal, piroplasm, schizont, signet ring

## Abstract

*Cytauxzoon felis*, a tick-borne hemoprotozoal pathogen of felids, causes an acute, often-fatal disease in domestic cats. While public awareness of the disease has increased, few studies have evaluated the incidence of acute cytauxzoonosis cases and their associated risk factors. The objective of this study was to retrospectively review records of cats diagnosed with acute cytauxzoonosis in eastern Kansas from 2006–2019 using clinic records and determine: (i) feline cytauxzoonosis risk factors; and (ii) if cytauxzoonosis case incidence is increasing. Although inter-annual variation of acute cytauxzoonosis diagnosis was observed in the eastern Kansas domestic cat population, the overall incidence trend remained largely unchanged over the 14-year case review period. In comparison to ill (*C. felis*-unrelated) control cases, more acute cytauxzoonosis cases were diagnosed in spring and summer, suggesting a seasonal fluctuation of infection, with samples most commonly submitted from ≥1 year old, owned, male cats. Although cytauxzoonosis case submissions remained consistent over the broad study period, increasing tick vector and domestic cat reservoir populations may contribute to additional cytauxzoonosis case expansion in endemic areas. Investigating the incidence of acute cytauxzoonosis, patient risk factors, and ecological variables that influence disease transmission are important steps towards developing and communicating the need for effective cytauxzoonosis control strategies for high-risk cat populations.

## 1. Introduction

**Figure 1:**
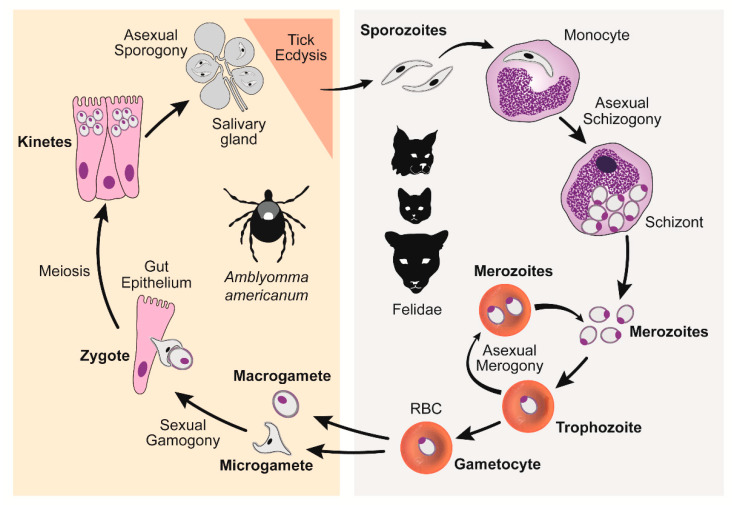
*C. felis* lifecycle. Right panel: *C. felis* reproduction within a felid host, asexual schizogony and merogony. Left panel: *C. felis* reproduction within a tick vector, sexual gamogony and asexual sporogony [10]. (Reproduced with permission from Yvonne Wikander, Pathogens; published by MDPI, 2020.).

Cytauxzoonosis is an often-fatal disease of domestic cats caused by *Cytauxzoon felis*, a tick-borne hemoprotozoal pathogen of felids. In the United States (U.S.), cytauxzoonosis cases most frequently occur in southeastern and south-central regions. This protozoal organism has a complex lifecycle that includes asexual reproduction in felid hosts and both asexual and sexual reproduction in competent ixodid tick vectors [1] (Figure 1). Briefly, *C. felis* sporozoites are transferred via tick saliva into a felid host during a blood meal. Once within the felid host, sporozoites enter monocytes and begin replicating asexually (schizogony) forming many 1–2 μm diameter signet ring merozoites. When the monocyte ruptures, these merozoites are released into the blood, where they enter host erythrocytes and begin either replicating asexually (merogony) or develop into gametocytes. Ticks become infected when they feed on an infected felid host and ingest gametocytes. The ingested gametocytes undergo sexual reproduction to form zygotes in the lumen of the tick gut. Zygotes invade the tick gut epithelium, transform into kinetes, and migrate to the salivary glands where they transform into sporozoites. Transstadial maintenance of the parasite through the larvae-to-nymph or nymph-to-adult ecdysis is required for the transmission of the infectious sporozoites to a new felid host during the subsequent tick bloodmeal. The competent biological transmission vectors of *C. felis* in the U.S. include *Dermacentor variabilis* (American dog tick) and *Amblyomma americanum* (Lone star tick) [2,3,4,5]. The range of *D. variabilis* encompasses the eastern U.S. as well as focal regions in the west [6,7]. The continuously expanding range of *A. americanum* covers the southeastern and mid-central U.S., largely overlapping with the range of *D. variabilis* [8,9]. Larval and nymphal *D. variabilis* prefer to feed on small to medium-sized mammals, whereas adults feed on larger mammals, including cats. In contrast, *A. americanum* are less discriminating, such that all life stages will seek out and feed on many mammals, including cats. Because of intensifying populations and its aggressive nature and willingness to feed on cats at all life stages, it is likely that *A. americanum* is a more significant vector of *C. felis* compared to *D. variabilis* in areas where both tick species reside.

Clinical cytauxzoonosis cases are most commonly observed in domestic cats (*Felis catus*), which serve as intermediate host carriers for *C. felis* along with other members of the Felidae family. Domestic cat cytauxzoonosis cases typically peak in late spring and early fall when the *A. americanum* adult and nymph life stages are most active [11]. For many decades, domestic cats were considered a dead-end host, as those with observed clinical disease commonly died [12,13,14]. Clinical disease signs for acute cytauxzoonosis appear during the schizogenous phase of *C. felis* asexual replication and can cause severe illness and death [2,12]. Cats commonly present with anorexia, depression, lethargy, pyrexia, dehydration, pale mucous membranes, and splenomegaly 11–13 days post infection (dpi) [2,15,16]. As the disease progresses, the pyrexia resolves and drops to subnormal, with icterus and dyspnea developing shortly thereafter. Complete blood count and serum biochemistry changes are seen around 13 dpi and can include variable cytopenias, hypoalbuminemia, hyperglycemia, and increased ALT (alanine aminotransferase) and total bilirubin [2,14,16]. Intra-erythrocytic signet rings (also known as piroplasms) and/or schizont-laden monocytes are often observed at the feathered edge of thin blood smear preparations [2]. The organs most affected include the lung, liver, spleen, and lymph nodes [13,14,16,17,18]. Death occurs around 21 dpi due to inflammatory cytokine-mediated injury of multiple organs, interstitial pneumonia, hypoxic injury, and disseminated intravascular coagulation [2,11,12,17,18,19,20]. If initiated early in the disease process, treatment for ten days with a combination of atovaquone (15 mg/kg) and azithromycin (10 mg/kg) may result in the recovery of up to 60% of patients [21]. For survivors, clinical signs begin to resolve around 23–24 dpi, after which they become subclinical infected carriers that act as reservoirs for further *C. felis* transmission via competent tick vectors [2,22]. Domestic cat carriers of *C. felis* may be more common than previously thought, as we recently identified *C. felis* carrier infection in 25% of cats in eastern Kansas [10]. Similar clinical signs and disease progression have been reported with *Cytauxzoon* spp. in South America, Europe, and Asia [23,24,25,26].

Only a few studies have examined the incidence and risk factors associated with acute cytauxzoonosis cases in U.S. domestic cats; however, the conclusions of these studies vary. For example, investigation into cytauxzoonosis incidence trends varied from stable [11] to increasing [27], while evaluation of specific risk factors varied from identifying a predisposition of disease in young male cats [16] to tick climate and habitat conditions being more predictive of the disease rather than cat age and gender [28]. Anecdotal reports of increasing acute cytauxzoonosis cases, as well as heightened public awareness of this disease, makes understanding cytauxzoonosis and associated patient risk factors important for mitigating disease transmission among high-risk cat populations. To address these concerns in eastern Kansas, the objectives of this study were to: (i) determine if acute cytauxzoonosis cases in eastern Kansas increased between 2006–2019; and (ii) examine if specific feline risk factors are correlated with acute disease or disease outcomes, by performing a retrospective records search and review of acute cytauxzoonosis cases submitted to the Kansas State Veterinary Diagnostic Laboratory (KSVDL). Based on the high density of *A. americanum* ticks in eastern Kansas and feline risk factors reported as important by cytauxzoonosis studies in other states, we hypothesized that cases of acute cytauxzoonosis would: (i) show an increasing trend over time; (ii) peak in seasons of peak *A. americanum* activity; and (iii) be reported more commonly in young, intact, male cats. Updated information on acute cytauxzoonosis incidence and risk factors will help identify and focus *C. felis* education and intervention efforts for high-risk domestic cat populations.

## 2. Materials and Methods

### 2.1. Ethic Approval

All study activities involving animals and management of animal data were performed in accordance with an approved Kansas State University Institutional Animal Care and Use protocol (IACUC 4109), approved prior to study initiation.

### 2.2. Study Design

A retrospective study with individual cats as the study subject was designed using case records from the Kansas State Veterinary Diagnostic Laboratory (KSVDL) in Manhattan, Kansas. The KSVDL case database was queried for the term “*Cytauxzoon felis*” among records between May 2006 and October 2019 using the laboratory management software VetView v1.6.12 (University of Georgia, Athens, GA, USA). All search-populated records were individually reviewed to confirm that the cases met study inclusion criteria—(i) domestic cats from eastern Kansas, and (ii) a confirmed diagnosis of cytauxzoonosis. Cases that came from within the Kansas State University teaching hospital, as well as outside submissions of blood and/or tissue samples, were included in the study. Exclusion criteria included: (i) any cat not from eastern Kansas; or, (ii) cases where cytauxzoonosis diagnosis was not confirmed. Data collected from each feline patient record included: specimen receipt date (year, month, season); geographic information (county, state); diagnostic method (necropsy vs. blood smear); blood smear diagnosis (schizont vs. signet rings); signet ring frequency (occasional vs. frequent); patient age (years); patient sex (male vs. female) and sterilization status (neutral vs. intact); feline lifestyle (stray, owned, or rescue/rescinded); patient live/dead status within 30 days of initial case record; and mention of similar household cat deaths in the medical record (yes vs. no). Historical data of 123 control cases from Raghavan et al. [28], which consisted of “cats with a history of fever, malaise, icterus, and anorexia but no *C. felis* on blood film examination or schizonts within macrophages from fresh tissue or within multiple organs”, were included as controls for this study [28].

### 2.3. Statistical Analyses

Yearly feline cytauxzoonosis incidence was analyzed under the Poisson log linear model with year block (2005~2009, 2010~1014, 2015~2019) being the fixed effect. Tukey’s multiplicity adjustment was applied when comparing incidence rates among year blocks. For assessing risk factors including age (<1 year, 1–3 years, 3–5 years, ≥5 years) and season (Winter (December, January, Febuary), Spring (March, April, May), Summer (June, July, August), Fall (September, October, November)), the present study data of 170 cases were combined with the previously published 123 non-cytauxzoonosis control cases [28]. The resulting case-control data gave rise to two-way contingency tables where the Pearson chi-square test for difference in risk factor distributions was applied. For assessing sex (male, female) and lifestyle (feral, owned, rescue/rescinded) risk factors; diagnostic method used (blood smear, necropsy); *C. felis* life stages observed (signet rings, schizonts); and the relative amount of signet rings observed (frequent vs. occasional), one-way tables were created from the study data. A chi-square goodness-of-fit test was then performed to compare the observed percentages against the hypothesized distributions. When the cell count(s) in a contingency table were less than five, *p* value was computed using the exact method instead of asymptotic approximation. The confidence interval for mortality rate was estimated using the Clopper–Pearson (exact) method. The confidence interval for percentage of owners of cats diagnosed with cytauxzoonosis reporting similar loss of other cats was estimated using the Wald method. Missing data were excluded from the statistical analyses. Statistical analyses were performed using the GENMOD and FREQ procedures of SAS/STAT^®^ software, Version 9.4 (SAS Institute Inc., Cary, NC, USA).

## 3. Results

### 3.1. Annual Acute Cytauxzoonosis Incidence in Domestic Cats from Eastern Kansas

**Figure 2:**
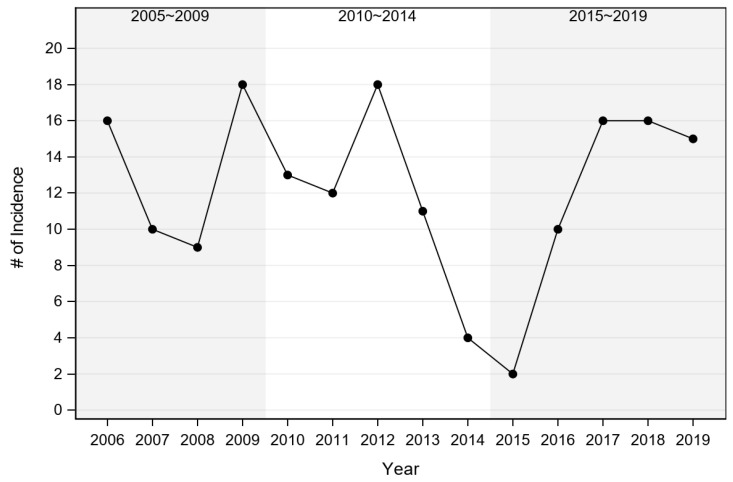
Incidence of acute cytauxzoonosis cases by year. Numbers represent raw acute cytauxzoonosis case counts.

A total of 183 domestic cat records with a diagnosis of acute cytauxzoonosis from May 2006 through October 2019 were identified. Thirteen records were excluded from the study because they had no identifiable state of origin (*n* = 3) or they came from adjacent states (*n* = 10), leaving records from 170 domestic cats diagnosed with cytauxzoonosis from eastern Kansas available for analysis. These 170 records were evaluated to determine if the incidence of acute cytauxzoonosis cases was changing over time. Of those cases with a reported live-dead status (*n* = 140), only one cat survived. The greatest numbers of acute cytauxzoonosis cases were reported in 2006, 2009, 2012, 2017, 2018, and 2019 (Figure 2, Table S1). Because the number of cases varied from year-to-year, testing results were evaluated by year blocks: 2005–2009, 2010–2014, and 2015–2019. No significant difference was detected between any of the year blocks (*p* = 0.754) (Table 1, Table S2). Although acute cytauxzoonosis inter-annual variation was observed over time, collectively the incidence was stable over a broader time scale.

**Figure 3:**
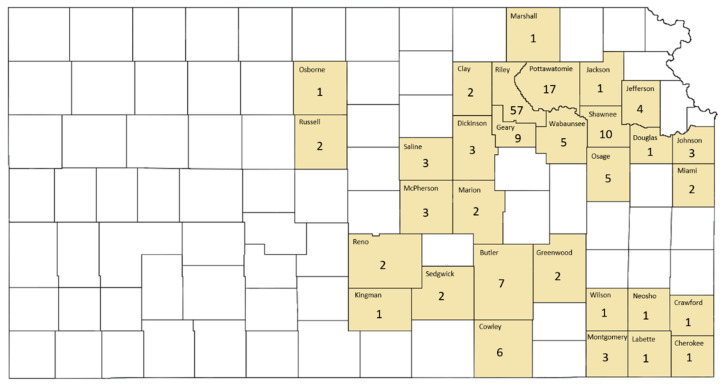
County-level location of acute cytauxzoonosis diagnosed domestic cat records in Kansas. Acute cytauxzoonosis case samples were submitted from cat(s) in shaded Kansas counties while no cat records were identified from unshaded counties. Numbers represent raw acute cytauxzoonosis case counts.

County-level locations of identified acute cytauxzoonosis cases in Kansas are presented in Figure 3. Acute cytauxzoonosis case samples were submitted to KSVDL from cats living in 31 eastern Kansas counties, with >50% of the samples coming from the county in which the KSVDL resides (Riley) and those immediately adjacent (Pottawatomie, Wabaunsee, Geary, Clay, Marshall). Statistical analysis of location data for acute cytauxzoonosis cases in specific counties was not possible because the sampling method across counties was not standardized.

### 3.2. Season Variation of Acute Cytauxzoonosis Incidence in Domestic Cats from Eastern Kansas

**Figure 4:**
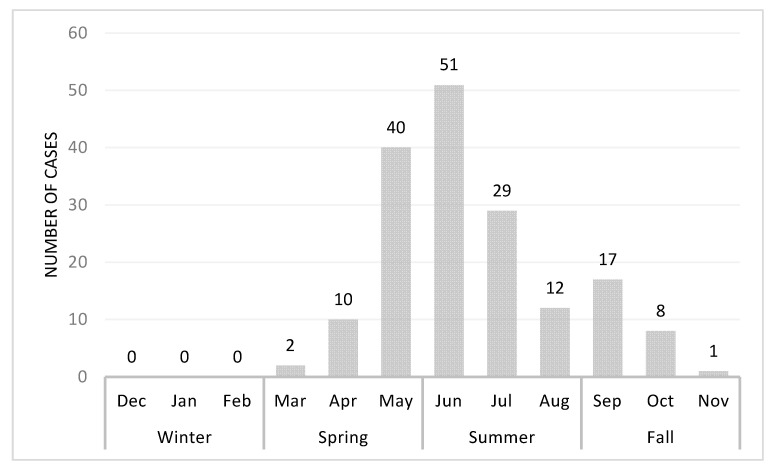
Acute cytauxzoonosis cases by month from May 2006 to Oct 2019. Case numbers represent the sum of total number of cases for each month from all years. These numbers represent raw acute cytauxzoonosis case counts.

To examine the intra-annual acute cytauxzoonosis case incidence, the incidence of acute cytauxzoonosis cases diagnosed in different months and seasons was evaluated (Figure 4). A bimodal distribution of cases was observed, with the greatest case peak between May (*n* = 40) and June (*n* = 51) and a second smaller case peak in September (*n* = 21). Similarly, the greatest proportion of acute cases was observed in summer (54.1%), then spring (30.6%) and fall (15.3%). The seasonal distribution of acute cytauxzoonosis cases was significantly different from that of ill (*C. felis*-unrelated) control case incidence (*p* < 0.001) (Table 2, Figure S1). The seasons that contributed most to the significance (i.e., large Pearson chi-square statistic) are fall with 15.3% for the acute cytauxzoonosis cases vs. 38.2% for the ill control cases, as well as winter with 0% for the acute cytauxzoonosis cases vs. 7.3% for the ill control cases.

### 3.3. Evaluation of Acute Cytauxzoonosis Incidence among Cats with Different Ages, Sex, and Lifestyle

**Figure 5:**
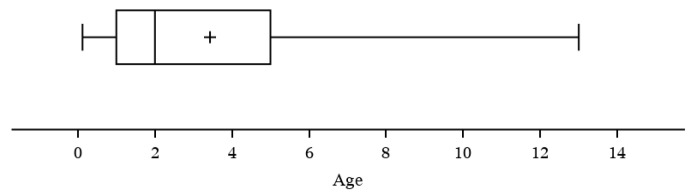
Acute cytauxzoonosis cases by age (years).

To examine if a correlation exists between specific cat risk factors with acute cytauxzoonosis case incidence, cat age, sex, and lifestyle were investigated. Feline age was reported in 156/170 cases evaluated, with the greatest number of acute cytauxzoonosis cases observed in cats 1–3 years old (40.4%) and with fewer cases observed in ≥5 years old (25.6%), 3–5 years old (21.8%), and <1 year old (12.2%) age groups (Table 3, Figure S2). The mean age of cats diagnosed with acute cytauxzoonosis was 3.4 years (range 1.4 months to 13 years) (Figure 5). The age distribution of acute cytauxzoonosis cases was significantly different from that of ill control case incidence (*p* < 0.001). The age group that contributed most to the significance (i.e., large Pearson chi-square statistic) is <1 year old with 12.2% for the acute cytauxzoonosis cases vs. 47.0% for the ill control cases.

Feline sex was reported in 162/170 cases evaluated, with a diagnosis of acute cytauxzoonosis observed significantly more in male (70.4%) compared to female (29.6%) cats (*p* < 0.001) (Table 4). The statistical analysis of sterilization status data was not possible as the sterilization status was not reported in the control group data [28] and data regarding sterilization status among the general Kansas cat population are unknown or unreported.

Acute cytauxzoonosis cases were predominantly owned cats (96.5%), with fewer feral and rescue/rescinded cats (2.4% and 1.2%, respectively) (Table 5). The distribution of lifestyle for acute cytauxzoonosis was significantly different (*p* < 0.001) to that for the general cat population of Kansas, with 49.9% feral, 47.1% owned, and 3.0% rescue cats [29,30,31,32]. Collectively, acute cytauxzoonosis was most commonly observed in ≥1 year old, male, owned cats.

### 3.4. Evaluation of Method Used to Diagnose Acute Cytauxzoonosis in Domestic Cats from Eastern Kansas

Lastly, the method used to diagnose acute cytauxzoonosis was evaluated (Table 6). Means of diagnosis did not vary significantly among acute cytauxzoonosis cases (*p* = 0.092); however, a small majority of cases were diagnosed via blood smear (56.5%) vs. via necropsy results (43.5%). Necropsy diagnosis was by visual identification of *C. felis* schizont(s) within vessels of any histological tissue sample. The tissues most commonly affected included the lung, liver, spleen, and lymph nodes. Blood smear diagnosis was made by visually identifying either the schizont (49%) or intra-erythrocyte signet ring life stages (51%) (also known as piroplasms) (Table 6). The blood smear diagnosis via schizont vs. signet rings did not vary significantly among acute cytauxzoonosis cases (*p* = 0.838). For those diagnosed through the identification of signet rings, the quantity of signet rings observed was generally classified as frequent (61.2%) or occasional (38.8%), which did not differ significantly from the 1:1 ratio (*p* = 0.116) (Table 6). Based on cases with a known disease outcome (1 survivor and 139 deaths), the survival rate in acute cytauxzoonosis was 0.7% with 95% confidence interval of (0.02%,3.92%). The sole survivor was diagnosed by schizont identification using the blood smear method.

## 4. Discussion

In *C. felis* endemic regions, acute cytauxzoonosis is one of the most fatal feline diseases. Several retrospective studies have demonstrated a bimodal pattern of seasonal cytauxzoonosis cases that correlates with *A. americanum* activity; young male cats being over-represented [8,11,16,27,28,33]. As a disease with a high fatality rate, increased public concern, and practitioner belief of increased incidence, understanding factors contributing to acute cytauxzoonosis can help focus efforts on preventing *C. felis* transmission among high-risk cat populations. In the present retrospective study, we demonstrate: (1) acute cytauxzoonosis case incidence in domestic cats from eastern Kansas has an overall stable trend; (2) acute cytauxzoonosis cases are more common in the spring and summer seasons; (3) young male cats are more likely to be diagnosed with acute cytauxzoonosis; and (4) schizont and/or intra-erythrocytic signet ring identification on histology or blood smear samples are reliable identifiers of acute cytauxzoonosis when associated with typical cytauxzoonosis clinical signs.

This study did not support anecdotal reports of an increasing acute cytauxzoonosis case trend in eastern Kansas cats based on KSVDL case sample submissions over the study period (Figure 2). Rather, the anecdotal reports of increasing acute cytauxzoonosis cases may be more attributed to individual year-to-year case fluctuations instead of a general increasing trend over the evaluated 14-year period (2006–2019). This finding agrees with a study that determined there was no significant year-to-year difference in 232 acute cytauxzoonosis cases identified over a 12-year period (1995–2006) in Oklahoma [11]. In contrast, another study determined there was an overall increase in acute cytauxzoonosis cases over a 10-year period (2001–2011) in western Kentucky [27]. However, the latter study consisted of only 56 cases and no statistical analysis method was reported. It is important to note that all three studies, including ours, were based on retrospectively evaluated data with limited patient details and histories. The discrepancy between anecdotal reports of increasing case numbers and data presented here may be due to a variety of reasons. Firstly, more clinicians may be diagnosing cytauxzoonosis with in-hospital microscopic blood smear evaluations versus sending out samples to a diagnostic laboratory, like KSVDL. Alternatively, an increased awareness of the disease may make the number of cases appear more prominent in the eyes of the clinicians, diagnosticians, and/or owners, while not actually being increased. Thirdly, vector tick populations—especially *A. americanum*—may be increasing or expanding their ranges with changing environmental conditions, increasing clinician index of suspicion and resulting in more suspected *C. felis* infection diagnoses without definitive diagnosis. Lastly, there may be an increasing incidence of acutely infected cats surviving infection without veterinary care, while the incidence of those presented to veterinary hospitals may be unchanged. Interestingly, with regard to this last point, we recently conducted a study investigating the prevalence of *C. felis* carrier (reservoir) cats in eastern Kansas and found that 25.8% of sampled domestic cats were actively infected with *C. felis* [10]. As such, more cats may survive acute cytauxzoonosis than previously expected, thus contributing to an expanding domestic cat reservoir population. The extent of *C. felis* genetic diversity and how these differences may affect virulence, transmissibility, and potential for treatment success is largely unexplored, with investigations hampered by a lack of methods to preserve or manipulate *C. felis* isolates outside of living cat or tick hosts. Attempts at identifying genetic markers of virulence have yet to find definitive virulence determinants [21,23,34,35,36].

A seasonal pattern of acute cytauxzoonosis incidence in domestic cats from eastern Kansas demonstrated a bimodal distribution, similar to those reported in other studies, peaking in early summer (June) with a smaller peak in early fall (September) [11,16,27,28]. This pattern corresponds with *A. americanum* peak activity in this area, which is predominantly dependent on environmental conditions like diurnal temperature range, precipitation, and humidity [8,28]. Adult ticks are most active in early spring to mid-summer, nymphs in late spring to mid-summer as well as late summer to early fall, and larvae in late summer to early fall [8]. This would suggest that *C. felis* may have been transmitted to the cats in this study predominantly by adults/nymphs in early summer with fewer transmissions occurring in early fall by nymphs. Previous tick transmission studies have demonstrated that both adult and nymphal *A. americanum* can successfully transmit *C. felis* [2,11]. However, since tick findings or tick preventative use practices were not identified in case records, further evaluation regarding the tick vector was not possible in this study. In addition, late spring through early fall may be times when cats spend more time outdoors actively roaming, resulting in a greater chance of encountering tick vectors. The combined seasonal cat and tick activity could explain why cats in our study area with evidence of fever, malaise, icterus, and anorexia were more likely to be diagnosed with acute cytauxzoonosis in spring/summer than other diseases with a similar presentation. A larger, long-range, prospective study evaluating both predisposing environmental conditions and habitat niches would provide insight into the timing of increased tick activity, which could be used to anticipate or forecast times of increased risk for *C. felis* transmission, as well as other tick-borne diseases.

As expected, young cats in their first and second year of life were more likely to have samples submitted and diagnosed with acute cytauxzoonosis. Our finding that young cats had a predisposition for acute cytauxzoonosis diagnosis supports previous data regarding age distribution for cats diagnosed with acute cytauxzoonosis [16] and tick infestation [33]. The first study, conducted in the mid-Atlantic states, found cats diagnosed with acute cytauxzoonosis (*n* = 34) had a mean age of 4 years (range 2 months to 14 years) [16]. Interestingly, another study investigating tick infestation of cats found cats with tick infestations were a mean age of 4.4 years (range 18 days to 18 years) (*n* = 336), with ticks recovered more frequently on young cats [33]. Combined, both studies support our study results of most commonly observing acute cytauxzoonosis cases in younger cats. In our study, a greater proportion of sample submissions came from young cats, for which there are several possible explanations. Firstly, young cats may explore their environment more aggressively and/or more frequently than older cats, resulting in potentially greater exposure to *C. felis* infected ticks. Secondly, owners may be more likely to present samples from younger cats with illness or an unexpected death, thinking an older cat died of old age. Thirdly, older cats may lack the energy or drive to return home when feeling ill and expire where their body is less likely to be retrieved. Lastly, older cats maybe be more likely to have encountered and survived a previous *C. felis* challenge, resulting in an asymptomatic reservoir stage. It is reasonable to assume that most cats that contract cytauxzoonosis do so through their outdoor activities because that is where the tick vector is most commonly located.

Among cats diagnosed with acute cytauxzoonosis in our study, male cats were over-represented (Table 4), supporting a previous study that also found male cats to be over-represented (20/31; 64.5% male cats in other study) [16]. Interestingly, male cats were also previously found to be more likely to have tick infestations than female cats (59% and 41%, respectively) [33]. At one time it was believed that male cats had territories up to ten times larger than female cats, which could explain these findings [37]. However, more recent studies determined there is no significant difference in home range sizes between male and female cats [38,39,40]. As such, it is unclear why male cats had a higher incidence of disease compared to female cats in our study and others. A larger sample size as well as more complete patient background information would have been helpful in more thoroughly evaluating acute cytauxzoonosis risk factors; however, we were limited by the information provided in the case records. Overall, our findings regarding acute cytauxzoonosis seasonality and feline age/sex predilections in Kansas provide additional support to age and sex being important risk factors.

Not surprisingly, most of the sample submissions in this study came from owned cats (96.5%), with rare rescue/rescinded or feral cat samples. Since owned cats generally benefit from a stronger human–animal bond, more diagnostics are performed either to understand the cause of a pet’s demise or to ascertain if a zoonotic or infectious potential exists for the owner and/or other household pets. Additionally, 26.5% (45/170) of the patient records evaluated included mention of multiple cats in the household dying of acute cytauxzoonosis or a cat that died in a similar but undiagnosed manner. These pet owners may be more sensitive to the potential for this disease in their pets, resulting in increased diagnostic efforts. Feral cats, barn cats, and/or outdoor “pets” which are fed but lack a strong human–animal bond with the owners are less likely to be submitted for diagnoses due to cost and/or difficulty in obtaining samples. This population of “wild” cats may also stray further afield, dying far from the owned property, or the property owners may be less attentive to these cats’ cause of death or disappearance. Rescue and humane society organizations have limited medical care budgets and are more likely to perform on-site diagnostics for treatment decisions, and less likely to submit tissue samples to an outside diagnostic laboratory after death.

**Figure 6:**
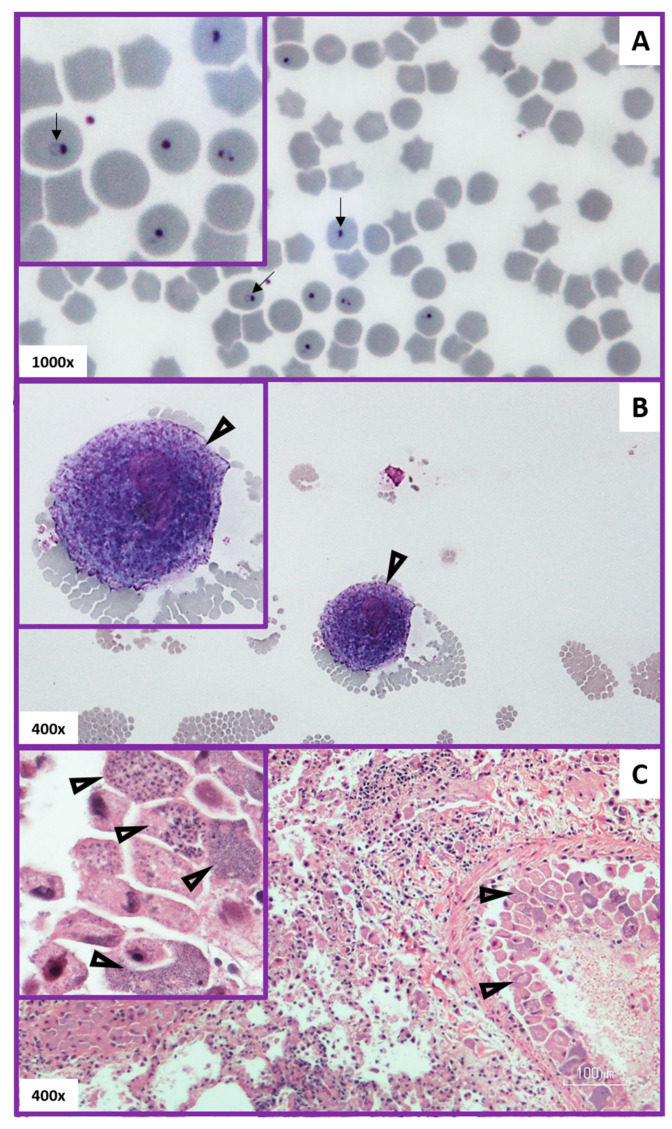
Diagnostic pathology of acute cytauxzoonosis. Panel (**A**) (top): Blood smear with intra-erythrocytic signet rings (arrows). Panel (**B**) (middle): Blood smear with a schizogenous monocyte (schizont) at the feathered edge (open arrowheads). Panel (**C**) (bottom): Histologic lung tissue with schizonts attached to endothelium (open arrowheads) within a vessel. Note the variable schizont developmental stages (arrows) seen in the Panel C inset.

Rapid, patient-side diagnostics are not currently available for cytauxzoonosis diagnosis. Instead, the diagnosis of acute cytauxzoonosis is most commonly accomplished by a review of blood smears by a clinical pathologist or a review of tissue histology after necropsy. A diagnosis of acute cytauxzoonosis via identification of *C. felis* schizonts or intra-erythrocytic signet rings in blood smears, or schizonts in histology tissue samples, was equally diagnostic (Figure 6). Additionally, the density of intra-erythrocytic signet ring forms seen in blood smears did not determine the severity of this disease as nearly all cats in this cohort died or were euthanized (99.3%) due to their illness. Different methods and criteria have been used in studies to diagnose acute cytauxzoonosis, including the identification of: (i) schizonts only [28]; (ii) schizonts or intra-erythrocytic signet rings [27]; or (iii) either schizonts or intra-erythrocytic signet rings with expected clinical signs [11]. Schizonts, when identified, are specific for acute cytauxzoonosis because this is the first life stage that develops in the feline host after the tick inoculates the sporozoites (Figure 1). In this study, observation of schizonts was noted in all case records where histologic tissue samples were evaluated (Figure 6C). That said, tissue sampling for histologic evaluation is far more costly and invasive than obtaining a blood sample, and requires a sedated, anesthetized, or deceased patient. Additionally, it does not demonstrate/confer a diagnostic advantage over a blood smear sample. Indeed, schizonts are not always identified on blood smear evaluation (Figure 6B). Approximately half of our blood smear samples lacked schizont identification. Piroplasms, 1–2 μm diameter intra-erythrocyte signet rings, are found in both the schizogenous and erythrocytic phase of cytauxzoonosis and are too small to be reliably seen on histology (Figure 6A). Blood smear cases lacking schizonts in this study had either multiple intra-erythrocytic signet rings per erythrocyte (active merogony), a high density of intra-erythrocytic signet rings, or occasional intra-erythrocytic signet rings in patients exhibiting clinical signs consistent with acute cytauxzoonosis. These presenting cats were unlikely to be merely a *C. felis* reservoir, which can also have low parasite loads (generally 0.045–1.27% infected erythrocytes), because they also exhibited clinical signs of cytauxzoonosis [22]. In subacute and early acute infections, intra-erythrocytic signet rings and schizonts may not be immediately apparent on blood smears. Blood or tissue sample submissions to a diagnostic laboratory for pathologist evaluation takes time that these patients may not have. Ideally, a rapid chairside test for in-house veterinary use would be developed to identify these cases very early in the disease process, allowing for early treatment initiation that may prove lifesaving for many of these cats.

The differential diagnosis for the nonspecific clinical signs seen in cytauxzoonosis (lethargy, fever, icterus, dyspnea, +/− anemia) could include cholangiohepatitis, triaditis, pancreatitis, hepatic lipidosis, sepsis, immune-mediated hemolytic anemia, toxins causing oxidative damage (e.g., *Allium* spp,, acetaminophen), neoplasia, and infectious agents (tularemia, feline infectious peritonitis, hemotropic mycoplasma), to name a few [41,42]. When intra-erythrocytic hemoparasites with a signet ring or piroplasm appearance are seen in cats, there are three main differential diagnoses: (i) *Cytauxzoon felis*, (ii) *Mycoplasma hemofelis*, and (iii) *Babesia felis*. *C. felis*, discussed herein, typically presents with a variable non-regenerative anemia, intra-erythrocytic signet rings, rare to occasional signet rings in the background, and/or schizonts at the feathered edge of blood smears or on histology of tissue samples. *M. hemofelis* commonly presents with a strong regenerative anemia, and signet ring and/or coccoid forms of epierythrocytic organism located on the red cells and/or in the background [41]. *B. felis* presents with a regenerative anemia and intra-erythrocytic signet rings often arranged in tetrads [42]. Considering our study area is within the U.S., *B. felis* is unlikely to be the cause of infection in these cats as *B. felis* has not been reported in the U.S. However, it should be considered for any feline patient with a travel history to Africa, particularly the southern coastal regions [42]. While *M. hemofelis* and *Babesia* spp. PCR is readily available for differentiation, *C. felis* PCR is less available. That said, identification of either schizonts and/or intra-erythrocytic signet rings via histology or blood smear samples are equally diagnostic for acute cytauxzoonosis when associated with typical acute cytauxzoonosis clinical signs for cats living in or near endemic areas. In locations where these feline piroplasm diseases overlap, DNA sequencing may be necessary to resolve piroplasm species identity.

## 5. Conclusions

In conclusion, we determined the incidence of acute cytauxzoonosis in domestic cats of eastern Kansas has remained stable over the last 14 years, commonly occurs in young male cats, and correlates with the expected *A. americanum* activity and life cycle. Clinicians practicing in or near endemic areas should consider acute cytauxzoonosis as a differential for any cat exhibiting depression, lethargy, fever, anorexia, icterus, anemia, cytopenias, or sudden death with confirmation via histologic or blood smear identification of schizonts or intra-erythrocytic signet rings and/or PCR. Since no vaccine exists and effective treatment options are limited, *C. felis* tick transmission should be mitigated by the use of aggressive, year-around, acaricide products for all domestic cats living in endemic areas, regardless of age, sex, or lifestyle. More studies are needed to further elucidate factors affecting cytauxzoonosis disease progression and presentation, and treatment options and outcomes within the U.S. and globally.

## Figures and Tables

**Table 1:** Statistical analysis of year block effect on acute cytauxzoonosis incidence.

	^1^ of Incidence per Year		Ratio to (Adj. *p* Value of Testing for Ratio ≠1)	
Year Block	Mean	SE ^2^	2010–2014	2015–2019
2005~2009	13.3	1.8	1.14 (0.764)	1.12 (0.813)
2010~2014	11.6	1.5	-	0.98 (0.995)
2015~2019	11.8	1.5	-	-

^1^ Number. ^2^ Standard Error.

**Table 2:** Statistical analysis of season effect on acute cytauxzoonosis and control case incidence.

		Case		Control		
Effect	Season	Count	Percent	Count	Percent	*p* Value of Exact Chi-Square Test
Season	Spring	52	30.6%	21	17.1%	<0.001
	Summer	92	54.1%	46	37.4%	
	Fall	26	15.3%	47	38.2%	
	Winter	0	0.0%	9	7.3%	

**Table 3:** Statistical analysis of age effect on acute cytauxzoonosis and control case incidence.

		Case		Control		
Effect	Age Group ^1^	Count ^2^	Percent	Count	Percent	*p* Value of Chi-Square Test
Age	<1	19	12.2%	47	47.0%	<0.001
	1–3	63	40.4%	24	24.0%	
	3–5	34	21.8%	18	18.0%	
	≥5	40	25.6%	11	11.0%	

^1^ Years, ^2^ Age data were available and evaluated from 156/170 case records.

**Table 4:** Statistical analysis of sex effect on acute cytauxzoonosis case incidence.

		Case			
Effect	Sex	Count ^1^	Percent	Percent Tested against	*p* Value of Chi-Square Test
Sex	F	48	29.6%	50.0%	<0.001
	M	114	70.4%	50.0%	

^1^ Sex data were available and evaluated from 156/170 case records.

**Table 5:** Statistical analysis of lifestyle effect on acute cytauxzoonosis case incidence.

		Case			
Effect	Lifestyle	Count	Percent	Percent Tested against	*p* Value of Exact Chi-Square Test
Lifestyle	feral	4	2.4%	3.0%	<0.001
	owned	164	96.5%	47.1%	
	rescue	2	1.2%	49.9%	

**Table 6:** Statistical analysis of diagnostic method, blood smear diagnosis, and relative number of signet ring form effects on acute cytauxzoonosis case mortality.

		Case			
Effect	Group	Count	Percent	Percent Tested against	*p* Value of Chi-Square Test
Method of Diagnosis	blood smear	96	56.5%	50%	0.092
	necropsy	74	43.5%	50%	
Blood smear rings vs. schizonts	rings	49	51%	50%	0.838
	schizonts	47	49%	50%	
Relative number of rings observed	frequent	30	61.2%	50%	0.116
	occasional	19	38.8%	50%	

## Supplementary Materials

The following are available online at https://www.mdpi.com/2306-7381/7/4/205/s1, Table S1: Acute cytauxzoonosis case incidence by year and year block, Table S2: Acute cytauxzoonosis case incidence by year block, Figure S1: Acute cytauxzoonosis and control case percentages by season, Figure S2: Acute cytauxzoonosis and control case percentage by age group (years).
