# Supplementary material for: Acute Cytauxzoon felis Cases in Domestic Cats from Eastern Kansas, a Retrospective Case-Control Study (2006–2019)
Source: Vet Sci. 2020 Dec 18;7(4):205. doi: 10.3390/vetsci7040205 (PMC7767035; doi:10.3390/vetsci7040205)
Supplement: Supplementary file 1 [file vetsci-07-00205-s001.pdf]

Table S1: Acute cytauxzoonosis case incidence by year and year block.

Year block	Year	# of Incidence per year
**2005-2009**	2006	16
	2007	10
	2008	9
	2009	18
	**Total**	**53**
**2010-2014**	2010	13
	2011	12
	2012	18
	2013	11
	2014	4
	**Total**	**58**
**2015-2019**	2015	2
	2016	10
	2017	16
	2018	16
	2019	15
	**Total**	**59**

Table S2: Acute cytauxzoonosis case incidence by year block.

Year Block	Total incidence #	Average incidence # per year
**2005~2009**	53	13.3
**2010~2014**	58	11.6
**2015~2019**	59	11.8

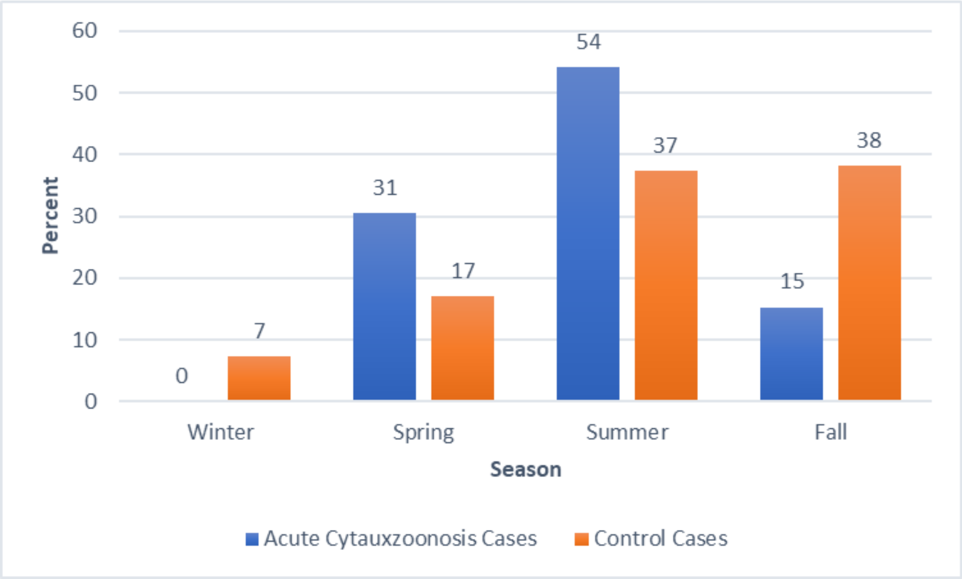

Figure S1: Acute cytauxzoonosis and control case percentages by season. The number above the bar represents the percent of cases by season.

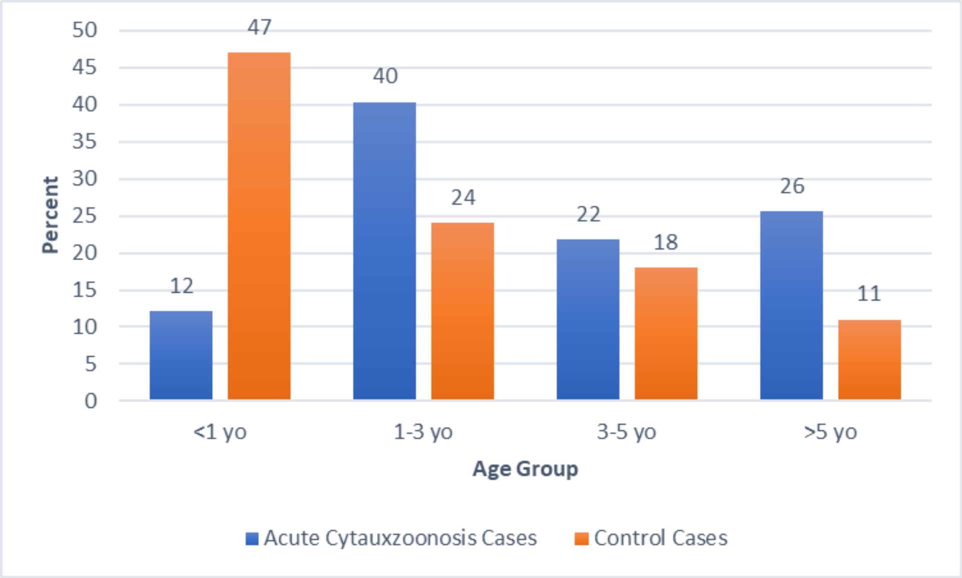

Figure S2: Acute cytauxzoonosis and control case percentage by age group (yo = years old). The number above the bar represents the percent of cases within a given age group.
